# DIFFERENT RESPONSE TO EXPOSURE TO AIR POLLUTANTS IN GIRLS AND BOYS

**DOI:** 10.1590/1984-0462/;2019;37;2;00009

**Published:** 2019-04-08

**Authors:** Renata Armani de Moura Menezes, Drielle Rezende Pavanitto, Luiz Fernando Costa Nascimento

**Affiliations:** a Universidade de Taubaté, Taubaté, SP, Brazil.

**Keywords:** Respiratory diseases, Child health, Fine particulate matter, Air pollutants, Doenças respiratórias, Saúde da criança, Material particulado fino, Poluentes do ar

## Abstract

**Objective:**

Identify the association between exposure to fine particulate matter and hospitalizations due to respiratory diseases in children up to ten years of age in the city of Cuiabá, Mato Grosso, stratifying the analysis by sex and calculating excess costs.

**Methods:**

Ecological study of time series. The dependent variable was daily hospitalizations according to the 10^th^ Revision of the International Classification of Diseases (ICD10): J04.0, J12.0 to J18.9, J20.0 to J21.9 and J45.0 to J45.0. The independent variables were the concentration of fine particulate, estimated by a mathematical model, temperature and relative air humidity, controlled by short and long-term trends. Generalized additive model of Poisson regression was used. Relative risks, proportional attributable risk (PAR) and excess hospitalizations and their respective costs by the population attributable fraction (PAF) were calculated.

**Results:**

1,165 children were hospitalized, 640 males and 525 females. The mean concentration, estimated by the mathematical model, was 15.1±2.9 mcg/m^3^ for PM_2.5_. For boys, there was no significant association; for girls a relative risk of up to 1.04 of daily hospitalizations due to respiratory diseases was observed for exposure to PM 2.5 in lags 1, 2 and 6. Increase of 5 µg/m^3^ in these concentrations increased the percentage of the risk in 18%; with an excess 95 hospital admissions and with excess expenses in the order of US$ 35 thousand.

**Conclusions:**

Significant effect in daily hospitalizations due to respiratory diseases related to exposure to fine particulate matter was noted for girls, suggesting the need for stratification by sex in further studies.

## RESUMO

**Objetivo:**

Identificar associação entre exposição ao material particulado fino e internações por doenças respiratórias em crianças de até dez anos de idade, no município de Cuiabá, MT, estratificando a análise por sexo e calculando excesso de custos.

**Métodos:**

Estudo ecológico de séries temporais, sendo a variável dependente o número diário de internações por doenças respiratórias, segundo a 10^a^ Revisão da Classificação Internacional de Doenças (CID): J04.0, J12.0 a J18.9, J20.0 a J21.9 e J45.0 a J45.0. As variáveis independentes foram a concentração do particulado fino, estimada pelo modelo matemático, além de temperatura e umidade relativa do ar, controladas pelas tendências de curta e longa duração. Foram calculados riscos relativos, risco atribuível proporcional (RAP) e excessos de internações e seus respectivos custos pela fração atribuível populacional (FAP).

**Resultados:**

Foram internadas 1.165 crianças (640 meninos e 525 meninas). A concentração média estimada do particulado fino foi 15,1±2,9 mcg/m^3^ para particulado fino (PM_2,5_). Para meninos, não houve associação significativa. Para meninas, observou-se risco relativo (RR) de até 1,04 para o número diário de internações por doenças respiratórias em associação à exposição ao PM_2,5_ nos *lags* 1, 2 e 6. O aumento de 5 mcg/m^3^ nessas concentrações associou-se ao aumento do risco em 18% e o RAP atribuído à exposição foi de 20% das internações das meninas, com excesso de 95 internações e de gastos da ordem de R$ 105 mil.

**Conclusões:**

Houve associação da exposição ao particulado fino e número de internações por doenças respiratórias de meninas, sugerindo a necessidade de estratificação por sexos em estudos posteriores.

## INTRODUCTION

In 2012-2013, about 50 thousand hospitalizations for respiratory diseases were registered in the state of Mato Grosso - five thousand in the capital, Cuiabá; children hospitalization, in particular, corresponding to 18 thousand in the state and 1,800 in the capital. These hospitalizations cost approximately R$ 13.6 million for the state and R$ 2.4 million for the city.^1^

In addition to known factors - such as low birth weight, lack of breastfeeding, overcrowded household, and presence of smokers -, exposure to air pollutants is also associated with hospitalizations for respiratory diseases, especially pneumonia.^2^ ^,^ ^3^ ^,^ ^4^ ^,^ ^5^ Particulate matter is among these pollutants. Particulate matter is a mixture of liquid and solid particles suspended in the air, whose composition and size depend on the emission source.^6^ It can be divided into two groups: one with particles with a diameter between 2.5 and 10 µm, called coarse fraction, and another with particles with a diameter smaller than 2.5 µm, called fine particulate matter (PM_2.5_).^6^ Fine particulate matter is important because it stays suspended for longer, can travel farther from their originating source, and, due to its diameter, reaches deeper parts of the respiratory system.^7^ Some studies have identified an association between exposure to fine particulate and hospitalizations for respiratory diseases.^8^ ^,^ ^9^ ^,^ ^10^ ^,^ ^11^ ^,^ ^12^ ^,^ ^13^

Air pollutants are usually quantified in measuring stations of state environmental agencies. However, not all states have environmental agencies, including Mato Grosso. An option is using mathematical models that estimate the concentrations of air pollutants, such as the Coupled Chemistry Aerosol and Tracer Transport model to the Brazilian developments on the Regional Atmospheric Modeling System (CCATT-BRAMS), which considers atmospheric dynamics. CCATT-BRAMS is an operational real-time monitoring system that uses the transport model. The Center for Weather Forecasting and Climate Studies of the National Institute for Space Research (*Centro de Previsão de Tempo e Estudos Climáticos/Instituto Nacional de Pesquisas Espaciais* - CPTEC/INPE) uses this model on an operational basis. The model estimates PM_2.5_ concentrations every three hours, resulting in eight estimates made at 40 m above the ground, with a resolution of 25 × 25 km.^14^ Current application of data estimated by CCATT-BRAMS can be found in epidemiological studies by Ignotti et al.,^15^ César et al.,^16^ Silva et al.,^4^ Nascimento et al.^17^, and Carmo et al.^18^

Recently, different responses to exposure to air pollutants according to the gender of adult subjects have been revealed. Studies suggest that health responses to air pollution could differ between women and men, and between girls and boys, but it is not yet clear whether the response observed is a result of biological differences related to gender or differences in activity patterns, co-exposure, or even accuracy in exposure measurement. Possibly, these differences consist of some combination of two factors - exposure patterns and biological response. ^19^ ^,^ ^20^ ^,^ ^21^

The objective of this study was to identify the effects of exposure to fine particulate matter on hospitalizations for respiratory diseases in children from Cuiabá, capital of Mato Grosso, state in the Amazon Region with a high number of fire outbreaks and which does not have an environmental agency, using data estimated by the CCATT BRAMS mathematical model, in addition to calculating the excess hospitalization costs.

## METHOD

**Figure 1:**
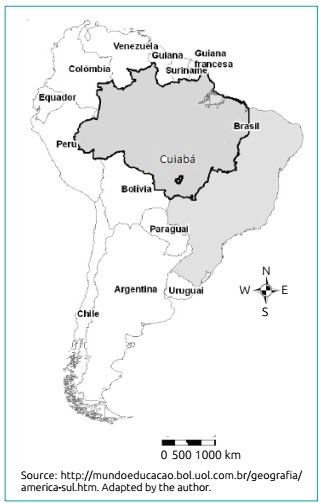
Location of the city of Cuiabá, MT, highlighting the Amazon Region.

We conducted a study on the hospitalization of children younger than ten years who live in the city of Cuiabá, capital of Mato Grosso, in the Midwest region of the country, which has a population of around 600 thousand inhabitants, at the coordinates 15°36’S and 56°5’O (Figure 1). The Technology Department of the public health system (*Departamento de Informática do Sistema Único de Saúde* - DATASUS)^22^ provided data on hospitalization (ICD 10, J04.0, J12.0 to J18.9, J20.0 to J21.9, and J45.0 to J45.9), and the National Meteorological Institute in Cuiabá, on minimum temperature and relative humidity. Data on PM_2.5_ pollutants were estimated by the CCATT-BRAMS model, developed by CPTEC/INPE. We created a time-series from January 1, 2012 to December 31, 2013 and recovered data on hospitalizations from November and December 2013 by researching information from January and February 2014.

The approach adopted was the generalized additive model of Poisson regression using the software Statistica^®^ (StatSoft, Inc., Tulsa, OK, USA), as data on the number of hospitalizations are discrete values and have Poisson distribution; the coefficients provided by this regression were transformed into relative risk (RR). The significance level of the study was alpha=5%. We carried out a descriptive analysis of these variables and, due to a difference in the effect of exposure over the days, we used a lag of up to seven days (lag 0 to lag 7). Relative risks were estimated with an increment of 5 µg/m^3^, using the expression RR=exp[b*5], in which b is the coefficient provided by the Poisson regression. With the value obtained from this increase in RR of hospitalization, we calculated the proportional attributable risk (PAR) with the expression PAR=[1-1/RR].

Next, we determined the population attributable fraction (PAF) given by the expression PAF=[PAR*N], in which n is the total outcome - in this study, the number of hospitalizations. The mean hospitalization cost in Brazilian reais was also calculated for the period under study, according to selected diagnostics.^21^ To find the value of potential excess costs, we multiplied the numerical result obtained for the PAF by the mean cost, in Brazilian reais, of hospitalization for respiratory diseases provided by the DATASUS website.

## RESULTS

In the study period, 1,165 children were hospitalized - 640 boys and 525 girls. Table 1 presents the mean, minimum, and maximum values - with the respective standard deviations - of the variables PM_2.5_, average temperature, relative humidity, and total and stratified (males and females) hospitalizations. In the study period, PM_2.5_ exceeded the limit accepted by the World Health Organization (WHO) (25 µg/m^3^) on 15 occasions. These values were found predominantly in September of both years and in isolated events in June 2012 and October and December 2013.

**Table 1:** Descriptive analysis of variables with mean daily values and respective standard deviation, and minimum and maximum values, Cuiabá, MT, 2012-2013.

	Mean±SD	Minimum-maximum
Fine particulate (µg/m^3^)	15.1±2.9	12.0-32.1
Humidity (%)	70.6±12.9	33.5-96.0
Minimum temperature (ºC)	21.1±3.2	8.5-27.8
Total hospitalizations (n=1,167)^#^	1.6±1.6	0-10
Boys hospitalized (n=640)^#^	0.9±1.1	0-8
Girls hospitalized (n=525)^#^	0.7±0.9	0-5

SD: standard deviation; ^#^total hospitalizations in the period.

Table 3 shows the Poisson regression coefficients, with their respective standard errors in parenthesis, provided by the model for the particulate matter, according to the gender of patients. It is noteworthy that, for males, no lag was statistically significant; on the other hand, females exposed to PM_2.5_ presented a risk factor in lags 1, 2, and 6; and there was no statistical significance for the entire year. Increasing 5 µg in PM_2.5_ concentrations raised the percentage risk for lags 1 and 2 or lag 6 by 18%, and the PAR accredited to exposure corresponded to 20% of hospitalizations of girls.

**Table 2:** Pearson correlation matrix of the variables: fine particulate, relative humidity, temperature, boys, girls, and total hospitalizations, Cuiabá, MT, 2012-2013.

	PM_2.5_	RH	TEMP	Boys	Girls	Total
PM_2.5_ (µg/m^3^)	1.00	-0.29^#^	0.08	-0.04	0.04	-0.01
RH (%)		1.00	-0.23	-0.01	-0.07	-0.03
TEMP (ºC)			1.00	-0.05	0.02	-0.02
Boys				1.00	0.22	0.80
Girls					1.00	0.76
Total						1.00

PM_2.5_: fine particulate matter; RH: relative humidity; TEMP: temperature; ^#^p<0.05.

Table 2 displays the Pearson correlation matrix and includes PM_2.5_, average temperature, relative humidity, boys and girls hospitalized, and total hospitalizations. We found an association between exposure and hospitalization for respiratory diseases only among girls, in lags 1, 2, and 6.

**Table 3:** Values of the Poisson regression coefficients and respective standard errors, in parenthesis, for boys, girls, and total sample, according to 0 to 7 lag days, Cuiabá, MT, 2012-2013.

	Boys	Girls	Total
Lag 0	-0.009612 (0.016144)	0.021744 (0.015106)	0.005138 (0.011011)
Lag 1	0.005279 (0.015310)	0.029924 (0.014864)	0.014186 (0.010658)
Lag 2	-0.006091 (0.015837)	0.036688 (0.013756)	0.013332 (0.010378)
Lag 3	0.008231 (0.014617)	0.002013 (0.016560)	0.004043 (0.010963)
Lag 4	-0.001658 (0.015991)	0.002294 (0.017832)	-0.001606 (0.011914)
Lag 5	-0.021042 (0.017211)	0.015908 (0.017713)	-0.003566 (0.012341)
Lag 6	-0.019364 (0.017637)	0.039610 (0.016117)	0.009629 (0.011883)
Lag 7	0.003688 (0.015526)	0.018130 (0.017243)	0.010643 (0.011532)

Bold: p<0.05.

**Figure 2:**
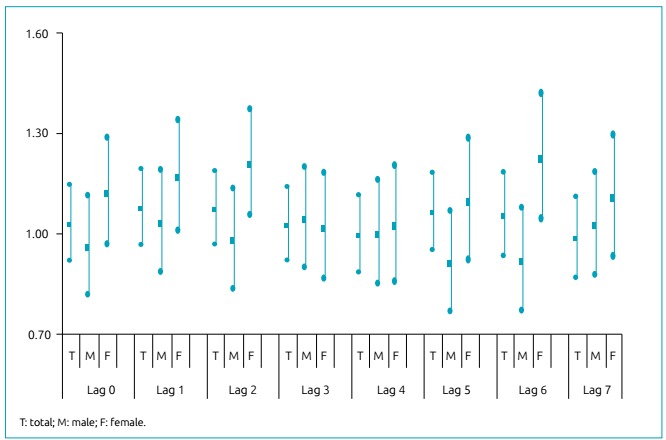
Relative risks of children hospitalization for exposure to fine particulate matter, according to gender (male and female) and total sample, Cuiabá, MT, 2012-2103.

Figure 2 illustrates the relative risks of hospitalization with the increase of 5 µg/m^3^, according to gender and total sample. RR reached a value of up to 1.22. This increase was responsible for 95 hospitalizations of girls, with a mean cost of R$ 1,100.00, resulting in an excess cost of approximately R$ 100 thousand (»$35,000.00).

## DISCUSSION

This 2-year study conducted in Cuiabá, MT, on children hospitalization for respiratory diseases identified an association between exposure to fine particulate matter and increased risk for girls in lags 1, 2, and 6. The effects manifested earlier - lags 1 and 2 - than in other studies.

Brazil has few studies that estimate the association between exposure to fine particulate and its effects on human health. A systematic review on the acute effects of exposure to fine particulate matter in Latin America found seven articles that related exposure to increased risk for respiratory disease or mortality with increments of 10 µg/m^3^ in concentrations of fine particulate matter for all ages.^23^

Brazil also has few studies that use the CCATT-BRAMS model to analyze the effects of PM_2.5_ exposure on the respiratory system, such as those developed by Cesar et al.^8^ and Patto et al.^10^ in cities where burning sugarcane straw is common, and by Silva et al.^4^ and Ignotti et al.^15^ in the Amazon Region. In addition to these studies, Cesar et al.^16^ in Taubaté, SP, and Nascimento et al.^11^ in Vitória, ES, analyzed the effect of fine particulate in regions where slash-and-burn of large proportions are uncommon. The values of PM_2.5_ concentration (15.0 µg/m^3^) identified in the present study were higher than those found in Vitória, ES,^11^ (11.4 µg/m^3^) and in another study conducted in Cuiabá, MT,^4^ with limits between 7.5 and 11.9 µg/m^3^, according to season - rainy or dry. However, the values of this research were lower than the ones found in Piracicaba, SP,^16^ (28.6 µg/m^3^) and close to those of São José do Rio Preto, SP, (18.7 µg/m^3^).^10^

Regarding RR of hospitalizations for respiratory diseases associated with exposure to fine particulate matter estimated in this study, females presented RR=1.04, a higher value than the one from Piracicaba - RR=1.02 -, despite Piracicaba having higher concentrations of fine particulate matter, but without stratification by gender. In a study performed in cities in the north of Mato Grosso, where PM_2.5_ concentrations were around 30.6 and 44.5 µg/m^3^ in the dry season, with a maximum of 260 µg/m^3^, the risk of hospitalization increased in up to 6% with the increment of 10 µg/m^3^ in concentrations of fine particulate matter.^24^ On the other hand, Andrade Filho et al.^25^ found no positive association between exposure to fine particulate and children hospitalization in the city of Manaus, AM, possibly due to the linear regression analysis adopted, with exposure being significant only for relative humidity rates.

In Cuiabá, with fine particulate data estimated by CCATT-BRAMS, the risk of children hospitalization for respiratory diseases was up to 22% in the dry season,^4^ coinciding with our values regarding lag days. A study also conducted in the Amazon Region identified toxicological risks from exposure to ozone, but not to fine particulate, whose concentrations reached 50 µg/m^3^.^12^

A Chinese study carried out by Duan et al.,^13^ with 2,200 hospitalizations that occurred in 2013, identified harmful exposure to fine particulate in lags 0 to 5, with a subsequent increased risk of hospitalization after an increment of 10 µg/m^3^ in concentrations of fine particulate, whose mean value was 150 µg/m^3^. In this study,^13^ the proportion of boys hospitalized was higher than that of girls, but the effects were similar in both genders, although more evident in males. It is noteworthy that the Chinese study used a multi-pollutant model, that is, the analysis included the pollutants particulate matter, sulfur dioxide, nitrogen dioxide, ozone, and carbon monoxide.^13^

There is strong evidence that exposure to fine particulate can lead to a health problem. These particles include sulfates, nitrates, acids, metals, and carbon particles with various chemicals adsorbed on their surfaces. Compared to the coarse fraction of particulate matter (particles with a diameter between 2.5 and 10 µm), the fine particulate penetrates more easily inside the house, travels longer distances, and due to its size, it can reach deeper lung structures. A possible route of action could involve oxidative lung injury and inflammation, with decreased pulmonary function, respiratory disorders, and cardiovascular diseases likely related to hypoxemia; alternatively, exposure to fine particles could cause alveolar inflammation, resulting in the release of potentially harmful cytokines and increased blood coagulability.^7^

The reason for the different responses to exposure to air pollutants between girls and boys is still not well established. A hypothesis concerns the inflammatory response, which would be more intense in girls with acute respiratory diseases. Women have a protective advantage in acute conditions, despite seeming more prone to deleterious tissue damage in relation to chronic inflammation, as in cystic fibrosis.^26^ On the other hand, during the H1N1 influenza pandemic, a study performed in Hong Kong showed that boys younger than 18 years tended to have higher rates of hospitalization for seasonal influenza than girls, which corroborated previous findings in Canada and Denmark, as well as the WHO report.^27^

These gender differences in the burden of hospitalization associated with influenza could be the result of distinctions between boys and girls regarding host susceptibility, risk of exposure, health-seeking behaviors, and immune response to vaccination against influenza and antiviral therapies.^27^ Boys tend to be more active than girls and have a higher risk of exposure to environmental pathogens (including influenza) through close contact with infected people or touch of contaminated surfaces. Previous studies also indicated that boys have a different immune response against influenza infections when compared to girls, but this difference gradually disappears as they grow.^27^

In addition, a study on pneumonia in children under one year of age carried out in South Africa revealed that boys had a higher incidence of the disease than girls in the multivariate analysis. The authors suggested the possibility of differences in intrinsic immune or inflammatory responses or differences in lung structure or function.^28^

Using biological markers, such as C-reactive protein, neutrophil count, and erythrocyte sedimentation rate, Casimir et al.^29^ identified striking differences between boys and girls under ten years of age, with higher values for girls hospitalized for pneumonia and bronchiolitis. Girls underwent longer hospitalizations and duration of fever, suggesting that gender can modulate the clinical expression of certain symptoms and, perhaps, the severity of the disease. These differences could be related to sex hormones, which can play a role not only in the synthesis control of growth hormone in early childhood but also in the management of acute inflammation. These authors suggest that increasing the inflammatory response could be beneficial in some pathological conditions, in which the acute inflammatory response could aid the full recovery.^29^

Limitations of this study could be in the analysis, which used data estimated by mathematical modeling, notwithstanding the validation of these estimated data for fine particulate. Another possible limitation is the lack of information about the living conditions of these children, the presence of smokers in the household, low birth weight, and breastfeeding. A third potential limitation relates to data, which did not account for hospitalizations by sources other than the public health system, such as private health insurance, in addition to outpatient treatment. We collected hospitalization data from an official source - DATASUS -,^22^ which has been systematically used in this type of approach. It is worth mentioning that the data presented in this study show an association and not causality.

Even with these plausible circumscriptions, we could identify the role of exposure to fine particulate matter in the hospitalization of girls from Cuiabá, MT, which suggests that this type of stratification should be adopted in further studies, as the analysis involving both genders was not significant.
